# An MRI-based classification scheme to predict passive access of 5 to 50-nm large nanoparticles to tumors

**DOI:** 10.1038/srep21417

**Published:** 2016-02-19

**Authors:** Anastassia Karageorgis, Sandrine Dufort, Lucie Sancey, Maxime Henry, Samuli Hirsjärvi, Catherine Passirani, Jean-Pierre Benoit, Julien Gravier, Isabelle Texier, Olivier Montigon, Mériem Benmerad, Valérie Siroux, Emmanuel L. Barbier, Jean-Luc Coll

**Affiliations:** 1 INSERM U823, Institut Albert Bonniot, Grenoble, France; 2 Université Joseph Fourier UJF, Grenoble, France; 3 Nano-H S.A.S., Saint Quentin – Fallavier, France; 4 INSERM U1066, IBS-CHU, Angers, France; 5 CEA-LETI MINATEC/DTBS, Grenoble, France; 6 Université Grenoble Alpes, Grenoble, France; 7 INSERM U836, Grenoble Institut des Neurosciences, Grenoble, France

## Abstract

Nanoparticles are useful tools in oncology because of their capacity to passively accumulate in tumors in particular *via* the enhanced permeability and retention (EPR) effect. However, the importance and reliability of this effect remains controversial and quite often unpredictable. In this preclinical study, we used optical imaging to detect the accumulation of three types of fluorescent nanoparticles in eight different subcutaneous and orthotopic tumor models, and dynamic contrast-enhanced and vessel size index Magnetic Resonance Imaging (MRI) to measure the functional parameters of these tumors. The results demonstrate that the permeability and blood volume fraction determined by MRI are useful parameters for predicting the capacity of a tumor to accumulate nanoparticles. Translated to a clinical situation, this strategy could help anticipate the EPR effect of a particular tumor and thus its accessibility to nanomedicines.

Low-molecular-weight targeted anticancer drugs administered intravenously are usually homogeneously distributed in most tissues but are expected to eventually perform their (specific) function in cancer cells only. Depending on the importance and quality of this specific therapeutic activity, these drugs often provide insufficient therapeutic benefits and cause severe systemic toxicity. In such cases, it is expected that their entrapment in a nanoparticle (NP) will reduce their accumulation in healthy tissues while improving it in tumors *via* the so-called “enhanced permeability and retention (EPR) effect^1^,^2^”. Indeed, molecules less than 40–45 kDa can leak out of the tumor vascular bed by diffusion, depending on the difference in concentration between the therapeutic solution and tumor. They are also rapidly cleared as they are evacuated by the lymph and blood circulation. By contrast, larger molecules and NPs (up to 500 nm in size) have greater difficulties extravasating from the vascular bed. They benefit from the augmented permeability of tumor blood vessels to leak out of the vascular bed more efficiently than in normal tissues under a convection flow, which can be represented by the difference in pressure between the therapeutic solution and tumor. Large molecules are then captured in the tumor’s interstitial space. The quantitative importance of the EPR effect is thus related to the tumor biology (*i.e.*, systolic blood pressure that pushes blood into the tumor tissue, blood and lymphatic vessel architecture and functions, interstitial fluid and extracellular matrix composition and pressure^3^,^4^,^5^, and the presence of tumor-associated cells and necrotic areas^6^), as well as to the physico-chemical properties of the NP’s (*i.e.*, stealth, circulation times, size, electrostatic charge, shape, and density^7^,^8^,^9^,^10^,^11^). The EPR effect is expected to increase the NP’s therapeutic index while reducing its toxicity. However, the number of clinical applications derived from this concept and number of nanovectors approved for human use remains limited^12^,^13^,^14^.

Altogether the intensity of the EPR effect remains extremely variable and unpredictable, and methods to estimate it in a given tumor to be treated are lacking. Thus far, predictive mathematical models are not practically applicable because too many parameters are measureless, ending up in an equation containing “black boxes^6^”.

We previously demonstrated that the combination of an iron-based steady-state vessel size index magnetic resonance imaging (VSI-MRI) approach using ultrasmall superparamagnetic iron oxide (USPIO) and a gadolinium (Gd)-based dynamic contrast-enhanced MRI (DCE-MRI) approach using Dotarem^®^ (Gd-DOTA) can be used to characterize the tumor microvasculature structure and permeability^15^,^16^,^17^. The present study investigated whether these clinically relevant measures could be used to anticipate how a given tumor can accept an NP, in a condition where there is no *a priori* knowledge of the physico-chemical properties of this NP.

The VSI- or DCE-MRI parameters were measured in eight different subcutaneous and orthotopic tumor models in mice. Next, optical imaging was used to evaluate if 5-nm or 50-nm large fluorescent NPs accumulate in the same tumors. Finally we evaluated whether the fluorescent signal could have been anticipated regarding the different MRI parameters taken separately or in combination. We demonstrate that it is possible to predict the capture of the fluorescent NPs in a given tumor based on two MRI parameters: the permeability and tumor blood volume fraction (BVf). This could help anticipate the capacity of a particular tumor to be “EPR sensitive”.

## Results

### Determination of the functional properties of the tumors using MRI

A total of thirty-five *nude* mice were engrafted with six different tumor cell lines, either with subcutaneous (SC) or orthotopic tumors (in the mammary fat pad with breast tumor cells or in the brain with glioblastoma cells). The chosen cell lines were of different origins and tumor stages (*i.e.*, IGROV1 (human ovarian carcinoma cells), HT29 (human tumor cells, known to produce well-differentiated adenocarcinomas, comparable to colonic primary carcinoma (grade I in mice), TS/a-pc (spontaneous and highly metastatic murine adenocarcinoma derived from ductal cells of the mammary gland), U87MG (human glioblastoma grade IV), HUH-7 (well-differentiated hepatocellular human carcinoma cells), and HEK293(ß3) (transformed normal human embryonic kidney cells).

**Figure 1:**
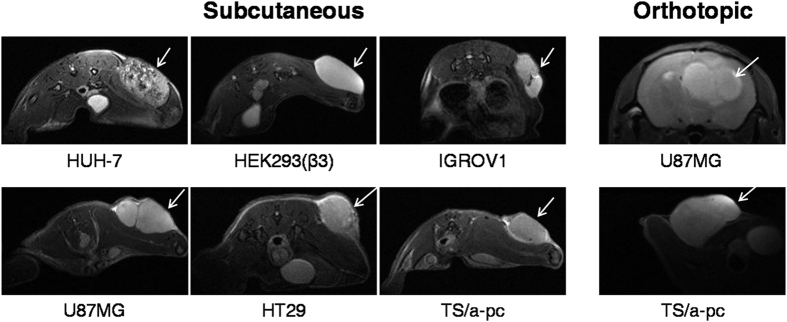
Anatomical views of the different tumor models. Six different tumor cell lines (HUH-7, HEK293(β3), IGROV1, U87MG, HT29 and TS/a-pc) were subcutaneously and/or orthotopically engrafted into mice. Anatomical coronal views of these tumor-bearing mice were obtained using a T2-weighted MRI sequence.

The anatomical view observed using a T2-weighted MRI sequence (Fig. 1) showed that HUH-7 tumors were heterogeneous, with large dark bloody areas. By contrast, the other tumor models were more homogenous throughout the entire tumor volume although small variations between adjacent nodules within the same tumor could be observed (as IGROV1 and U87MG examples).

**Figure 2:**
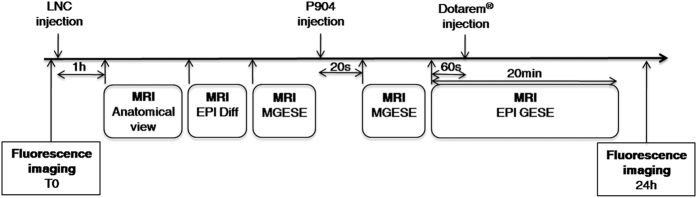
Diagram of the imaging experimental protocol. MRI anatomical view is obtained with a T2-weighted MRI sequence. LNC: lipid nanocapsule; P904: ultra small particle iron oxide (USPIO); EPI Diff: diffusion-weighted, spin-echo, single shot; MGESE: combined multi gradient-echo and spin-echo conventional MRI; EPI GESE: combined gradient-echo and spin-echo, single shot, EPI.

**Figure 3:**
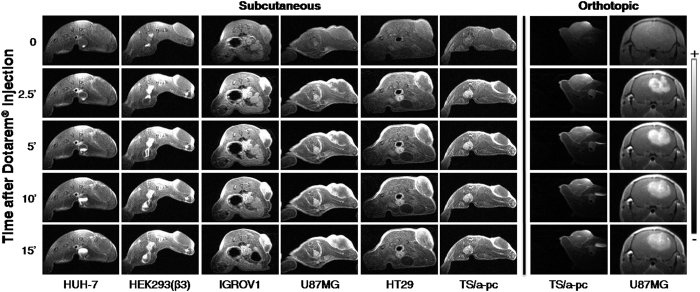
Evaluation of the permeability of the different tumors. Mice were imaged using a T1 MRI sequence immediately before (t = 0) and during the first 15 minutes after intravenous injection of Dotarem^®^. The hypersignal represents Dotarem^®^. Grayscale: 0–12,000.

Using a single MRI session (Fig. 2), it is possible to measure the tumor vessel’s permeability using Gd-DOTA (Dotarem^®^) and to measure the vessel size index (VSI) and tumor blood volume fraction (BVf) after the administration of ultra small superparamagnetic iron oxide (USPIO). The data obtained after the injection of Gd-DOTA permitted distinguishing among four types of tumors with variable levels in vessel permeability (Fig. 3 and Table 1). The HUH-7 tumors had a low permeability, with a low Gd signal after 2.5 minutes that decreased after 10 minutes. The TS/a-pc tumor vessels had heterogeneous permeability, for both subcutaneous and orthotopic tumors, as observed in HEK293(ß3) tumors. The strong Gd signal on the edge of the tumor suggested that there was an elevated permeability in the periphery of the tumor mass, whereas the rest of the tumor was impermeable. Tumors, such as U87MG or HT29, had an elevated permeability at their periphery 2.5 minutes after Dotarem^®^ injection; thereafter, the Gd diffused rapidly toward the interior of the tumor. Interestingly, the orthotopic U87MG tumors exhibited a fairly homogeneous contrast enhancement. Finally, IGROV1 tumors were very permeable, and a strong signal was observed in large domains of the tumor, with Gd still diffusing 15 minutes after the injection (Fig. 3).

The intravenous injection of USPIOs allowed the investigation of tumor blood vessels parameters (Supp. Fig. 1). Both the “vessel diameter” and “blood volume fraction” measures were calculated from the VSI-MRI sequences. The MRI sequences were performed 20 seconds after the USPIO injection. In this time frame, USPIOs do not extravasate from the blood flow. The HUH-7 and HEK293(ß3) tumors were characterized by a small number (~18 vessels/0.573 mm^2^) of “standard size” blood vessels (~12 μm in diameter) (Table 2). Others, such as U87MG and HT29 had an elevated number (>30 vessels/0.573 mm^2^) of “standard size” blood vessels while IGROV1 tumors were vascularized by a small number of larger blood vessels. TS/a-pc tumors presented very different configurations depending on their implantation site: numerous “standard size” vessels in SC tumors, or a smaller number of large-diameter vessels (~16 μm in diameter) when engrafted in the mammary fat pad. This trend was reversed when U87MG cells grew subcutaneously or orthotopically in the brain, suggesting that the physiological constraints in the organ of tumor development (*i.e.*, brain and mammary fat pad) impacted greatly on the organization of the neo-vasculature and thus on the EPR effect. We also evaluated the number of blood vessels and mean vessel diameter after anti-CD31 immunohistochemistry (IHC) staining of tumor sections (Supp. Fig. 2). High correlations were obtained when IHC and MRI results were compared (*p* < 0.001), thus validating the MRI measurements (Table 2).

### Passive accumulation of fluorescent nanoparticles

Fluorescent DID-containing 50-nm large lipidic nanocapsules (LNCs) were intravenously injected in the mice previously evaluated by MRI. These nanocapsules were distributed in the body with a blood half-life of 6.28 hours and a preferential hepatic elimination, as described in Basile *et al.*^18^ and summarized in the Supplementary Fig. 3A.

**Figure 4:**
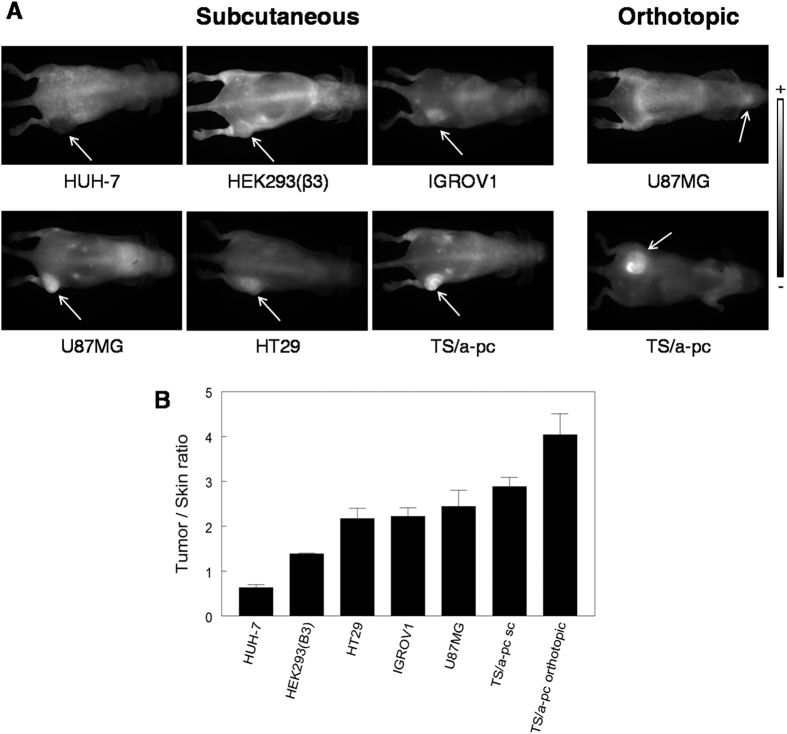
2D-fluorescence images of subcutaneous (left panel) and orthotopic (right panel) tumor-bearing mice 24 hours after LNC intravenous injection (10 nmol of dye). (**A**) Images were acquired over a period of 100 ms and are presented in the same grayscale (1,700–30,000). The arrows indicate the tumors. (**B**) Corresponding tumor/skin ratios 24 hours after LNC injection. Note that tumor/skin ratio was not accessible for orthotopic U87MG brain tumors.

LNC accumulation was visualized using 2D-FRI (Fig. 4) and semi-quantified on isolated organs (Supp. Fig. 4), as well as on whole-body images to calculate the tumor/skin ratio. These analyses were performed 24 hours after LNC injection; at this time point, the amount of LNCs in the tumor is representative of their kinetic of accumulation. The fluorescent signal of LNCs in tumors varied depending on the tumor model (Fig. 4 and Supp. Fig. 4B). Based on our experience of fluorescence in our imaging systems, an “EPR-negative” status was attributed to tumors that presented a tumor/skin ratio inferior to 1.5, corresponding to fluorescence intensity less than 5,000 photons/pixel/50 ms for this batch of fluorescent LNCs and for these experimental conditions. Thus, tumors such as IGROV1, HT29, TS/a-pc and U87MG, with elevated fluorescence intensities and tumor/skin ratios greater than 2, were considered “EPR-positive” tumors. By contrast, HUH-7 tumors and HEK293(ß3) were barely fluorescent and were considered as “EPR-negative” tissues (Fig. 4 and Supp. Fig. 4B). This confirmed our former results with HEK293(ß3), establishing also that the levels of LNCs in the tumors and in different organs were similar when measured by fluorescence or SPECT using dual-labeled DiD-^99m^Tc-LNC^19^.

### Prediction of LNC accumulation using the MRI measurements

We firstly tried to correlate each of the four parameters (tumor permeability, water diffusion coefficient, vessel diameter (VSI) or blood volume fraction (BVf)) with the fluorescent signal (Table 1). Taken separately, none of these parameters directly correlated with the fluorescence intensity (Supp. Fig. 5).

We next examined whether the different MRI parameters could be combined in a linear regression to establish a correlation with fluorescence as follows:


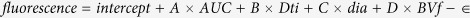


A correlation with the fluorescence intensity was possible only when using the permeability and vessel diameter parameters together (Supp. Fig. 6). However, although each parameter had a significant impact on the equation with a *p* value < 0.2, the total R^2^ value of 0.264 was not satisfying and confirmed the poor correlation between fluorescence and the different MRI parameters.

**Figure 5:**
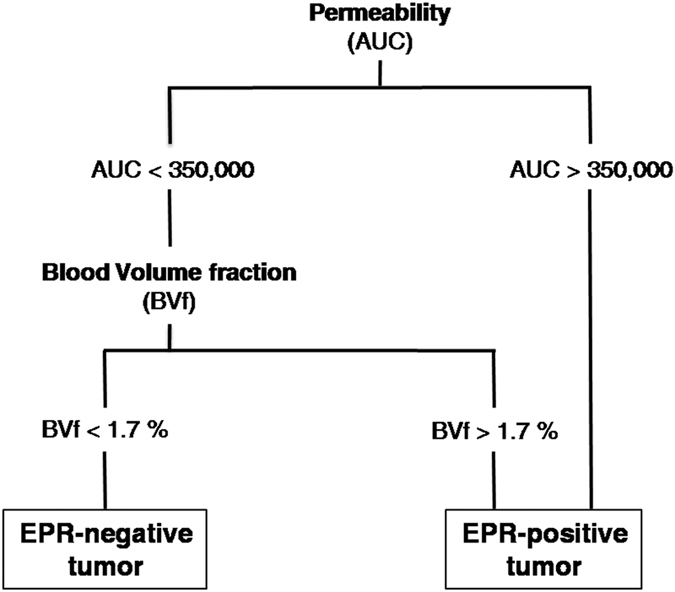
Determination of the EPR status according to the “classification scheme”. AUC: area under the curve; BVf: blood volume fraction.

Using another approach based on a “classification scheme”, we observed that combining “permeability” and “BVf” measures could perfectly predict whether a given tumor was accessible by the 50-nm large LNCs (Fig. 5). Indeed, “EPR-negative” tumors could be characterized by their permeability less than 350,000 (AUC) and BVf less than 1.7%. Using these two limits, the “EPR-negative” group (n = 6/35) and “EPR-positive” group (n = 29/35) were satisfyingly defined with a *p* < 0.0001. This finding indicated that tumors with a low permeability index and reduced blood access presented extremely poor passive EPR-mediated accumulation of the 50-nm large fluorescent LNCs.

### Confirmation of the tumor accumulation variability using two other types of nanoparticles

To confirm the differences in the ability of tumors to accumulate LNCs, two other types of nanoparticles were evaluated : lipidic nanoemulsions (LNEs) and ultrasmall rigid platforms (USRPs) (See Supp. Fig. 3A–C).

LNEs are 50-nm DiD-loaded lipidic nanoparticles, with chemical properties close to those of LNCs, although their elasticity and surface coating are different. After intravenous injection, LNEs were distributed in the body with a hepatic elimination and a mean residence time of 8 hours in the blood^20^ (Supp. Fig. 3B).

**Figure 6:**
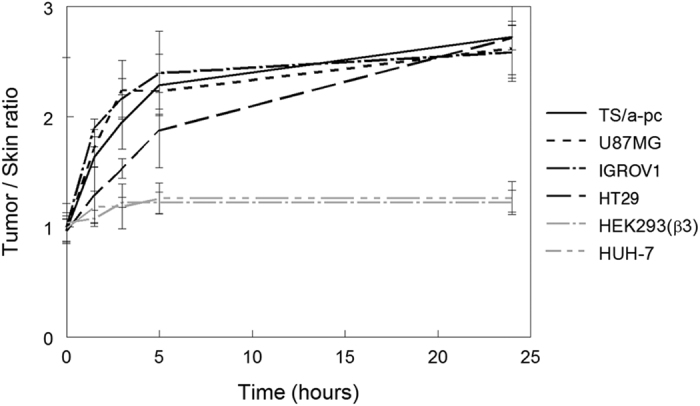
Passive accumulation of lipidic nanoemulsions (LNEs) in six different subcutaneous tumors after intravenous injection. The LNEs were intravenously injected (10 nmol of dye) into subcutaneous tumor-bearing mice (n = 3/group). 2D-fluorescence imaging was performed before and 1.5 hours, 3 hours, 5 hours and 24 hours after the injection of LNEs. Tumor and skin fluorescence signals were semi-quantified on whole-body images. The results are presented as the mean ± SD.

LNEs were injected into a set of mice bearing the six types of subcutaneous tumors previously studied (n = 3/group). The fluorescent signal was semi-quantified from whole-body images, and the tumor/skin ratios were calculated at different time points after intravenous injection of LNEs. In agreement with the data obtained with LNCs, the results showed that HUH-7 and HEK293(ß3) tumors exhibited tumor/skin ratios ranging from 1 to 1.5, corresponding to “EPR-negative” tumors. By contrast, the four other tumor types (*i.e.*, IGROV1, U87MG, HT29 and TS/a-pc) exhibited tumor/skin ratios greater than 1.5 as early as 3 hours after injection, reaching 2.5 at 24 hours (Fig. 6). These tumors can be considered “EPR-positive”.

**Figure 7:**
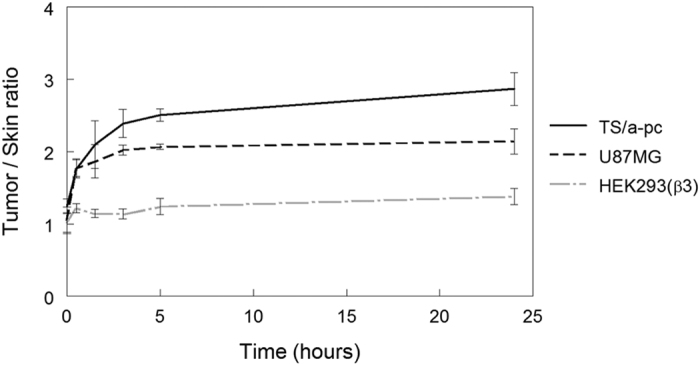
Passive accumulation of ultrasmall nanoparticles (USRPs) in three different subcutaneous tumors after intravenous injection. The USRPs were intravenously injected into subcutaneous tumor-bearing mice (n = 4/group). 2D-fluorescence imaging was performed before and 1.5 hours, 3 hours, 5 hours and 24 hours after the injection of USRPs. Tumor and skin fluorescence signals were semi-quantified on whole-body images. The results are presented as the mean ± SD.

Next, the ability of fluorescent cyanine5.5-USRPs to accumulate in tumors was evaluated in mice bearing subcutaneous HEK293(ß3) (n = 4), U87MG (n = 4), and TS/A-pc (n = 4). The USRPs are multimodal nanoparticles with a size less than 5 nanometers. They are composed of polysiloxane and gadolinium complexes covalently grafted to the inorganic matrix^21^. As indicated in Supp. Fig. 3C, the *in vivo* biodistribution of USRPs in healthy mice indicate that they have a blood half-life of 30 minutes and are eliminated exclusively by renal excretion^22^. As predicted, while the HEK293(ß3) tumors were presenting only background fluorescence similar to that of the surrounding healthy tissues (tumor/skin ratios ranging between 1.0 and 1.5), U87MG and TS/a-pc tumors were positively with tumor/skin ratios of 2.0 and 2.9, respectively. In these two last categories, the positive contrast was obtained as early as 1.5 hours after intravenous injection and is still detectable 24 hours later (Fig. 7).

## Discussion

Although it is quite simple to evaluate the ADME (*i.e.*, absorption, distribution, metabolism and excretion) of an NP, it is difficult to anticipate the physio-pathological fluctuations and inter-tumor variability that dictate how efficiently a given tumor in a given patient will be accessible or not to nanodrugs. We addressed this problem by following the distribution and passive EPR-mediated accumulation of intravenously injected 50-nm large fluorescent lipid nanocapsules in eight different subcutaneous or orthotopic tumor models engrafted into mice. The quality of the EPR-based tumor targeting was then compared with various functional parameters obtained by dynamic contrast-enhanced MRI (DCE-MRI) and vessel size index MRI (VSI-MRI).

Despite well-known limitations *i.e.,* the lack of anatomical information and poor penetration depth of light (requiring the use of near infrared dyes), the use of non-invasive fluorescence imaging has increased in the last decades^23^,^24^. This imaging tool is effective for monitoring the biodistribution of fluorescent NPs and visualizing inter-tumor variations of the passive accumulation in mouse-bearing tumor models. In this study, fluorescence imaging results clearly document the possible passive accumulation of two different types of 50-nm large NP (*i.e.,* LNCs and LNEs) in different tumor types and allow for the differentiation of “EPR-negative” and “EPR-positive” tumors. These results also confirm the biodistribution and whole pharmacokinetic measurements that were previously obtained with a similar batch of fluorescent-only^25^,^26^ or dual-labeled (fluorescent and radioactive) LNCs^19^.

Interestingly, similar variations of the passive tumor accumulation were obtained after injection of the very small USRPs (< 5 nm in size). Maeda *et al.*^2^,^13^,^27^ described that the EPR effect apply to NPs and macromolecules with an apparent molecular size larger than 40 kDa and a long blood circulation time (>6 hours). USRPs have a molecular size of 8.5 kDa and a blood half-life of 30 minutes, and thus, their passive accumulation cannot be formerly attributed to the EPR effect. However, USRPs were capable of passive accumulation in the same tumors initially classified as “EPR-positive” with the larger NPs. This is suggesting that the access and eventual positive accumulation of NPs is governed by the characteristics of the tumor itself. Indeed, interstitial blood pressure, vascular architecture, permeability, and diffusion coefficients are defining a threshold authorizing or not the access to the tumor. This inter-tumor variation represents a major problem that can occur in patients and prevent access of the drugs to some tumors or metastasis^28^. To a certain extent, the physico-chemical parameters of the injected NPs play minor roles at this level (at least for those NPs that are already acceptable for intravenous injection). It seems particularly important to develop methods, including imaging, to anticipate a possible passive accumulation^13^. Recently, a study demonstrated that the level of tumor vascularization, correlated with the EPR-mediated tumor accumulation of polymeric drug carrier, can be evaluated by contrast-enhanced functional ultrasound imaging^29^.

In this study, we used DCE-MRI (after injection of Gd-DOTA) and VSI-MRI (after injection of USPIOs), which can provide quantitative parameters of the tumor microenvironment in addition to tumor-specific vascular parameters abnormalities in preclinical cancer models^15^,^16^,^17^.

No direct correlation was found between the passive accumulation and the number and size of blood vessels, their permeability, quantity of blood or water diffusion coefficient. However, we established that the combination of “permeability” and “blood volume fraction” parameters enabled the prediction of whether the tested fluorescent NPs will accumulate or not in the different preclinical tumors. Importantly, this is not a prediction of the level of accumulation of the NPs, since this is definitely depending also on the NP’s pharmacokinetic properties, but rather on an ON/OFF type of response that is mainly related to the tumor characteristics.

Clinical studies also demonstrated the interest of DCE-MRI to differentiate between benign and malignant tumors^30^, assess the aggressiveness of tumors^31^,^32^ or predict the response to conventional chemotherapy^33^,^34^.

This approach may have a major impact on clinical practice. Indeed, these methods need to be validated in an appropriate cohort of patients to establish the optimal parameters to ensure the maximum distribution and retention in humans, which could differ from those required in animal models. Then a similar “classification scheme” could be determined in order to classify a given tumor as either “responsive” or “not-responsive”. A positive ranking suggests that nanomedicines will reach this particular tumor, whereas a negative one alerts the clinician to the possibility that the treatment may fail as a result of poor accessibility. Additionally, different strategies could be applied to augment the tumor responsiveness and overcome the heterogeneity of the EPR effect within tumor tissues^35^,^36^. These strategies could involve vascular-modulating agents such as angiotensin-II which induce hypertension^37^, angiotensin II-converting enzyme (ACE) inhibitors which enhance bradykinin levels and nitric oxide production^38^, nitric oxide-realizing agents^39^,^40^, and hyperthermia^41^. These treatments can enhance the blood flow and vascular permeability in tumors or open occluded blood vessels^42^.

## Conclusion

The present approach might be very useful in optimized and adaptive medicine based on the selection of patients using imaging, particularly MRI, for improved nanomedicine-based treatments.

## Methods

### Cell lines and culture conditions

The HUH-7 cell line, a well-differentiated cell line from a human differentiated hepatocellular carcinoma^43^ (kindly provided by M. Ozturk (INSERM U823)), was cultured in DMEM plus 10% FBS (fetal bovine serum; Invitrogen, Cergy-Pontoise, France). IGROV1 human ovarian adenocarcinoma cells^44^ (kindly provided by L. Poulain (Centre de Lutte contre le Cancer François Baclesse, Caen, France)) were cultured in RPMI 1640 medium supplemented with 10% FBS and 1% glutamine. U87MG human primary glioblastoma cells (ATCC^®^ HTB14^™^) were cultured in DMEM plus 10% FBS supplemented with non-essential amino acids. HT29 human colorectal adenocarcinoma cells (ATCC^®^ HTB-38^™^) were cultured in McCoy’s medium supplemented with 10% FBS. Finally, TS/a-pc mouse mammary carcinoma cells^45^ were cultured in RPMI 1640 supplemented with 1% glutamine and 10% FBS. HEK293(β_3_), stable transfectants of the human embryonic kidney cell line (kindly provided by J.-F. Gourvest, Aventis, France) were cultured in DMEM plus 10% FBS supplemented with 700 μg/ml Geneticin (G418 sulfate; Gibco, Paisley, UK). Of note, the original HEK293 cell line was obtained by the transformation of cultures of normal human embryonic kidney cells, and is not derived from a tumor. However, these cells form tumors when injected into *nude* mice. All of the cell lines were cultured at 37 °C in humidified 95% air/5% CO_2_ atmosphere.

### Lipid nanoparticles preparation

The lipidic nanocapsules (LNCs) and the lipidic nanoemulsions (LNEs) were prepared as described in Hirsjärvi *et al.*^26^. The two types of nanoparticles were loaded with the DiD (1,1′-dioctadecyl-3,3,3′,3′-tetramethylindodicarbocyanine perchlorate) near infrared fluorescent dye (Invitrogen, Cergy-Pontoise, France). The final local dye concentration in the nanocarriers was approximately 4.4 mM. The nanoparticles have a diameter of 50 ± 1 nm, and a PolyDispersity Index of 0.03 and 0.19 for LNCs and LNEs, respectively. The complete description of LNCs and LNEs characteristics and *in vivo* biodistribution are provided in Supplementary Fig. 3 A,B.

### Ultrasmall Nanoparticles

Ultrasmall rigid platforms (USRPs) were prepared as described previously^21^. They are composed of a polysiloxane network covalently grafted by gadolinium chelates [1,4,7,10-tetraazacyclododecane-1,4,7,10-tetraacetic acid (DOTA)-Gd]. They have a hydrodynamic diameter of 4.1 ± 1.0 nm and a zeta potential of −0.2 mV at pH = 7.4. An average of one cyanine 5.5 molecule was grafted for every 550 atoms of gadolinium. The description of USRPs characteristics and *in vivo* biodistribution are provided in Supplementary Fig. 3C.

### Animal studies

Animal studies were performed in strict accordance with French Ministry of Agriculture regulations and were conducted with protocols approved by both the Ethical Committee of Grenoble and the Grenoble Institute of Neurosciences Ethical Committee.

### ***In vivo*** experiments

Tumor-bearing mice were anesthetized using 4% isoflurane for induction and then at 2% isoflurane to maintain the respiratory rate between 50 and 70 breaths/min.

A catheter filled with saline was inserted into the mouse tail vein to enable the injection of a contrast agent comprising the following: 100 μl of LNC one hour before the beginning of the MRI studies, USPIO (P904; 250 μmol/kg, Guerbet, SA Aulnay-sous-Bois, France; apparent diameter of 25–30 nm; plasma half-life of approximately 62 min) and Gd-DOTA (200 μmol/kg; Dotarem^®^, Guerbet, France; apparent diameter of 1 nm, plasma half-life of approximately 26 min). Injections were always delivered manually and by the same investigator.

MRI was performed on a 4.7 T scanner (Biospec 47/40 USR AV III, Bruker, Germany – Grenoble MRI facility IRMaGE) equipped with a 12-cm inner diameter actively shielded gradient insert (640 mT/m in 120 μs). Actively decoupled volume and surface coils were used for excitation and reception, respectively (Bruker, Germany). Animals were placed in the prone position. All the images were acquired in the coronal orientation.

MRI sequences were composed of the following views: I) An anatomical view (T2-weighted) RARE-T2 MRI (TR = 4 s, TE = 33 ms, 1-mm-thick slices, matrix = 196 × 196). This sequence was merely used for tumor localization; II) Diffusion-weighted, spin-echo, single shot, EPI-64 (EPI Diff; TR = 5 s, TE = 43 ms, 1-mm thick slices, matrix = 128 × 128). This sequence was applied four times: once without diffusion weighting and three times with diffusion weighting (b = 800 s.mm^−1^) in three orthogonal directions; III) Combined multi gradient-echo and spin-echo conventional MRI (MGESE; TR = 3 s, sixteen echoes evenly spaced gradient-echoes (GE) = 3 to 83.1 ms, 1-mm-thick slices; matrix = 128 × 128). Immediately following this sequence, USPIOs were injected in 5 s; IV) A repetition of step III following a delay of 20 s; V) Combined gradient-echo and spin-echo, single shot, EPI (EPI GESE; TR = 710 ms, TE = 5.21 ms, 1-mm-thick slices, matrix = 128 × 128). This MRI sequence was applied repeatedly during 20 min (1 image/15 seconds). The bolus of 20 μl Gd-DOTA was injected 1 min after the beginning of this sequence over 2 s (flush of 70 μl of saline solution) (Fig. 2).

### Data processing

Data were processed in a MATLAB environment (v7.6; The MathWorks Inc., Natick, MA, USA). Regions of interest (ROIs) were manually slice-wise (1 mm thick) delineated for each mouse. The tumor and muscle ROIs were considered for each slide.

The tumor volume, water diffusion coefficient, blood volume fraction, vessel diameter, permeability and VSI were obtained for each slice. The results are expressed as the means of all the tumor slices for each mouse.

Image J^®^ software was used for images processing (grayscale).

### 2D-FRI imaging

Tumor-bearing mice were intravenously injected either with DiD-loaded LNCs (100 μl, [LNC] = 55 mg/ml)), DiD-loaded LNEs (200 μl, [LNE] = 50 mg/ml) or cyanine5.5-USRPs (200 μl, [Gd^3+^] = 10 mmol/L). Anesthetized mice were restrained in a prone position after induction and were illuminated by 660-nm light emitting diodes equipped with interference filters. Fluorescence reflectance images (FRI) were acquired over a period of 50 ms (for isolated organs) or 100 ms (for *in vivo* imaging), at different time points before and after injection of the NPs, with a back-thinned cooled charge-coupled device (CCD) camera (ORCAII-BT-512G, Hamamatsu Photonics, Massy, France) and a colored-glass RG9 high-pass filter (MellesGriot, Voisins le Bretonneux, France) to cut-off all excitation light. Semi-quantitative data were obtained from the fluorescence images of isolated organs by drawing regions of interest on the area to be quantified. The results are expressed as photons/pixel/50 ms. Quantification and analysis of photons recorded on the images were carried out using the WASABI image software (Hamamatsu Photonics).

### Immunohistochemical labeling

Tumor slices were placed at room temperature, fixed in acetone for 10 min and washed twice in TBS with 0.1% Tween 20 (TBS/T). Endogenous peroxidases were blocked with 1% H_2_O_2_/methanol, and slices were rinsed in distilled water and TBS/T for 5 min. Tumor sections were blocked with 2% rabbit serum diluted in TBS for 20 min and were rinsed for 5 min in TBS/T. CD31 was labeled with the rat anti-CD31 antibody for 1 hour (#550274 at 1/500; BD Biosciences, Pont de Claix, France) and the rabbit anti-rat antibody for 1 hour (#7077 at 1/750; Cell signaling Technology, Danvers, MA, USA). The labeling was detected with DAB for 5 min (#K3467; Dako, San Antonio, TX, USA) and rinsed with water for 5 min. Cell nuclei were stained with Gill’s hematoxylin for 2–3 s and washed with water 5 for min. Samples were dehydrated with 2 baths of 100% ethanol and one with xylene before the addition of mounting medium (M-GLAS, #1.03973.001; Merck Millipore, Fontenay sous bois, France). Images were taken using the Olympus BX-41 microscope with a 10× objective.

The evaluation of blood vessel number and diameter by CD31-quantification was performed on four fields of view for each tumor using Metamorph software.

### Statistics

The predictive equation, its different parameters and its cross validation were determined using a stepwise method in SAS^®^ software (SAS^®^ Institute, North Carolina, USA).

The predictability of the “classification scheme” was evaluated by a Fischer test. A Wilcoxon test was applied to compare the vessel diameters evaluated by MRI and IHC in tumor sections.

## Additional Information

**How to cite this article**: Karageorgis, A. *et al.* An MRI-based classification scheme to predict passive access of 5 to 50-nm large nanoparticles to tumors. *Sci. Rep.* **6**, 21417; doi: 10.1038/srep21417 (2016).

## Supplementary Material

Supplementary Information

## Figures and Tables

**Table 1:** MRI and fluorescence data obtained for each mouse.

		Mouse	Fluorescence	Permeability	Water Diffusion Coefficient	Vessel diameter	Blood Volume fraction
		#	mean (RLU/pixel/50 ms)	AUC	μm^2^/s	μm	%
Subcutaneous	HUH-7	1	689	98,522	998	12.41	2.17
		2	1,211	135,335	1,156	12.77	2.04
		3	1,580	139,081	1,219	14.55	3.16
	HEK293(β3)	1	4,659	420,034	827	14.48	1.28
		2	4,941	381,695	1,232	14.34	1.02
		3	4,790	269,158	879	10.54	1.25
	IGROV1	1	9,682	795,062	1,537	14.12	2.44
		2	12,505	519,448	1,495	13.05	2.86
		3	16,574	752,720	1,092	17.24	1.83
		4	10,288	569,030	1,356	15.86	2.81
	HT29	1	13,953	590,555	1,623	13.27	2.19
		2	11,062	361,986	1,762	14.47	2.22
		3	17,997	316,137	1,413	14.11	2.09
		4	6,252	350,655	1,216	12.69	1.66
		5	10,573	609,850	1,345	16.70	2.73
	TS/a-pc	1	23,076	820,437	1,073	14.06	3.14
		2	13,094	622,315	948	12.16	2.42
		3	9,761	302,853	965	11.94	2.41
	U87MG	1	12,935	365,001	1,150	11.47	1.79
		2	14,224	779,970	1,209	13.55	3.21
		3	8,943	521,711	1,142	13.51	2.14
Orthotopic	TS/a-pc	1	8,589	677,228	882	13.43	2.34
		2	10,311	677,525	1,292	15.67	2.67
		3	9,897	838,269	1,602	17.17	3.10
		4	19,681	447,632	1,479	18.14	3.68
		5	21,012	352,881	1,133	17.00	3.68
		6	21,471	452,775	1,539	16.60	2.68
	U87MG	1	10,151	677,864	798	13.19	6.17
		2	7,573	305,940	817	12.40	6.16
		3	8,891	414,801	783	16.60	8.40
		4	11,986	505,689	806	12.40	6.16
		5	15,298	430,359	880	13.91	7.92
		6	12,594	552,622	906	13.11	7.89
		7	12,197	720,627	884	12.98	6.15
		8	13,530	467,415	856	10.89	5.82

**Table 2:** Evaluation of the vessel diameter and number by MRI and IHC in tumor sections.

	Subcutaneous						Orthotopic	
	HUH-7	HEK293(β3)	IGROV1	U87MG	HT29	TS/A-pc	TS/A-pc	U87MG
MRI vessel diameter	13.24 ± 1.14	13.12 ± 2.23	15.07 ± 1.85	12.84 ± 1.19	14.25 ± 1.54	12.72 ± 1.17	16.34 ± 1.63	13.19 ± 1.63
IHC vessel diameter	14.53 ± 0.27	11.71 ± 1.60	15.08 ± 1.98	10.40 ± 1.18	11.52 ± 0.86	8.93 ± 0.75	16.43 ± 3.87	9.96 ± 0.7
number of vessels	17.1 ± 6.6	17.9 ± 6.4	18.4 ± 6.4	31.4 ± 6.4	34.5 ± 8.6	40.3 ± 17.2	14.41 ± 6.92	46.56 ± 12.7

The number of vessels (number of vessels per 0.573 mm^2^) and their diameters (in μm) were counted.These results were compared to vessel diameters (in μm) initially determined by MRI. The results were expressed as the mean ± SD.
