# Supplementary material for: An MRI-based classification scheme to predict passive access of 5 to 50-nm large nanoparticles to tumors
Source: Sci Rep. 2016 Feb 19;6:21417. doi: 10.1038/srep21417 (PMC4759815; doi:10.1038/srep21417)
Supplement: Supplementary Information [file srep21417-s1.pdf]

An MRI-based classification scheme to predict passive access of 5 to 50 nm large nanoparticles to tumors.

Anastassia Karageorgis[1, 2§,] Sandrine Dufort[1, 2, 3§], Lucie Sancey[1,2 §#], Maxime Henry[1, 2], Samuli Hirsjärvi[4], Catherine Passirani[4], Jean-Pierre Benoit[4], Julien Gravier[5, 6Δ], Isabelle Texier[5, 6], Olivier Montigon[2, 7], Mériem Benmerad[1, 2], Valérie Siroux[1, 2], Emmanuel L. Barbier[2, 7], Jean-Luc Coll[1, 2 *]

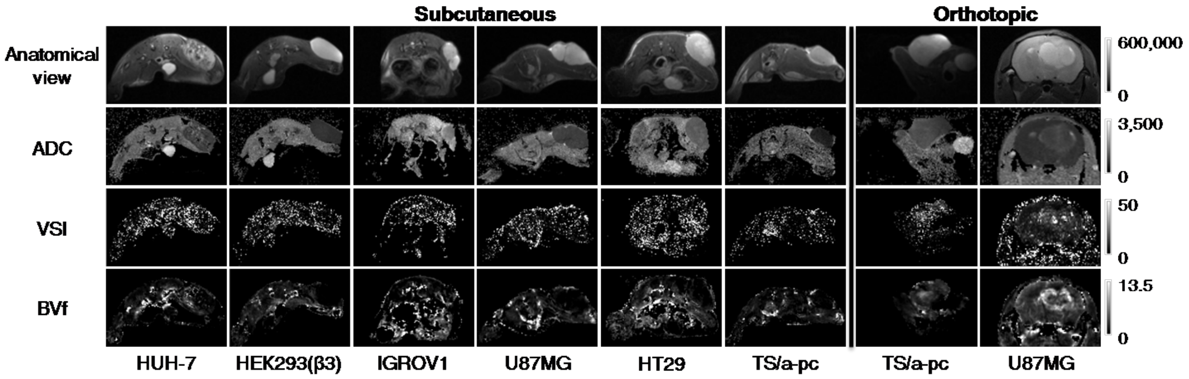

Supplementary Figure 1: Examples of images obtained with the different MRI sequences for each tumor model. Anatomical view (T2-weighted); Apparent Diffusion Coefficient (ADC) map; Vessel Size Index (VSI); Blood Volume fraction (BVf).

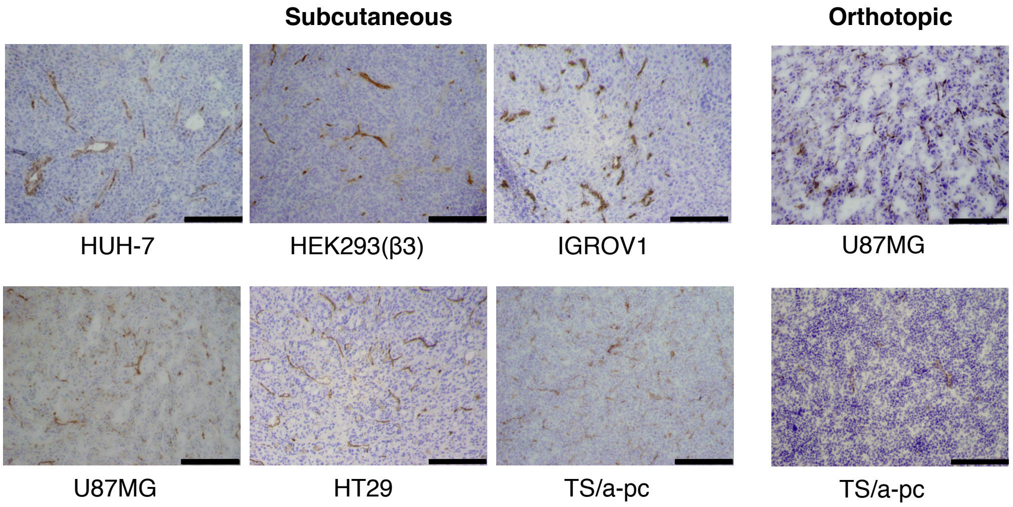

Supplementary Figure 2: Blood vessel immuno-staining performed on tumor sections using an anti-CD31 antibody (Scale bar 200 μm).

A

Main characteristics of LNC nanoparticles	
Size	50 ± 1 nm
Zeta potential	- 3 ± 1 mV
Blood half-life	6.28 hours

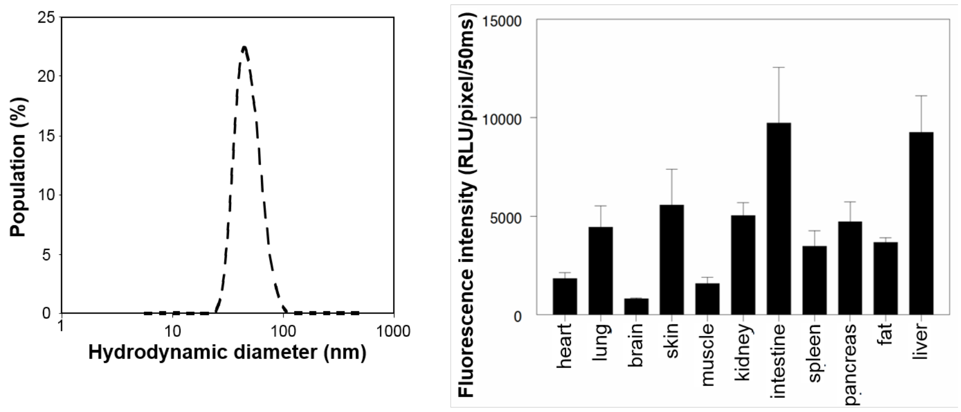

B

Main characteristics of LNE nanoparticles	
Size	50 ± 1 nm
Zeta potential	- 7 ± 1 mV
Blood half-life	8 hours

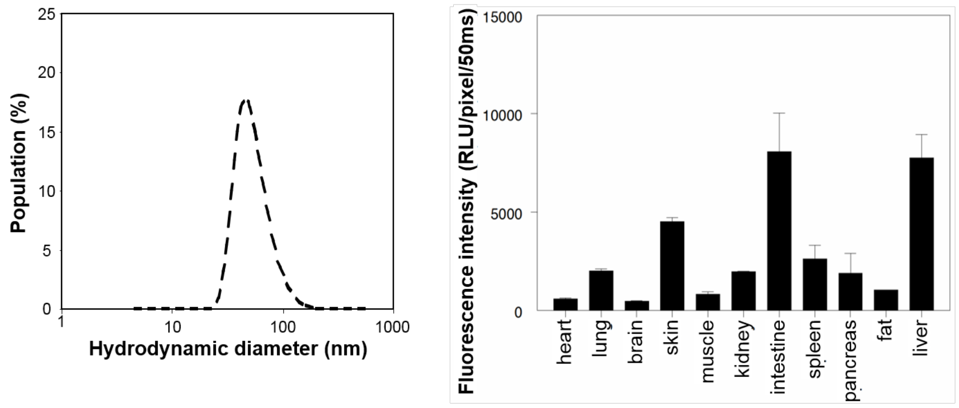

C

Main characteristics of AGuIX nanoparticles	
Size	3 ± 0.1 nm
Zeta potential	- 9.03 ± 5.5 mV
Blood half-life	30 minutes

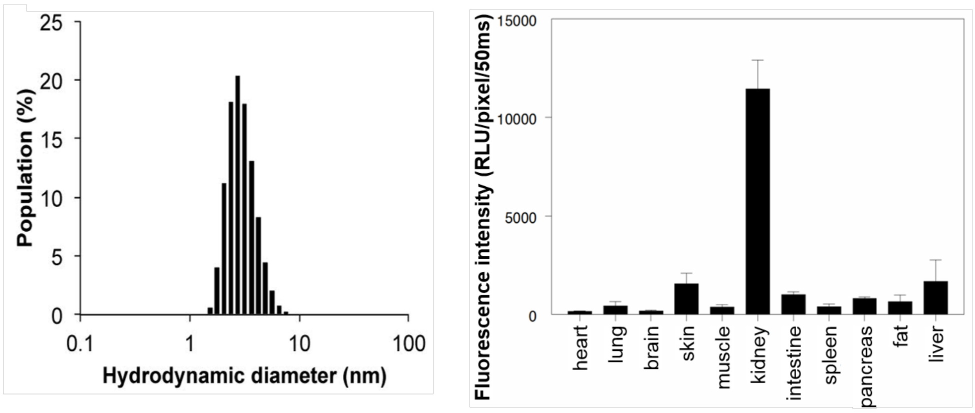

Supplementary Figure 3:

Characterization of the different nanoparticles' properties. In addition to the average size and hydrodynamic diameter, the electric charge is presented. Using fluorescence imaging after systemic tail vein administration, the blood half-life and tissue accumulation were measured in the mice's organs 24 hours after LNCs, LNEs or USRPs intravenous injections (10 nmol of dye).

A

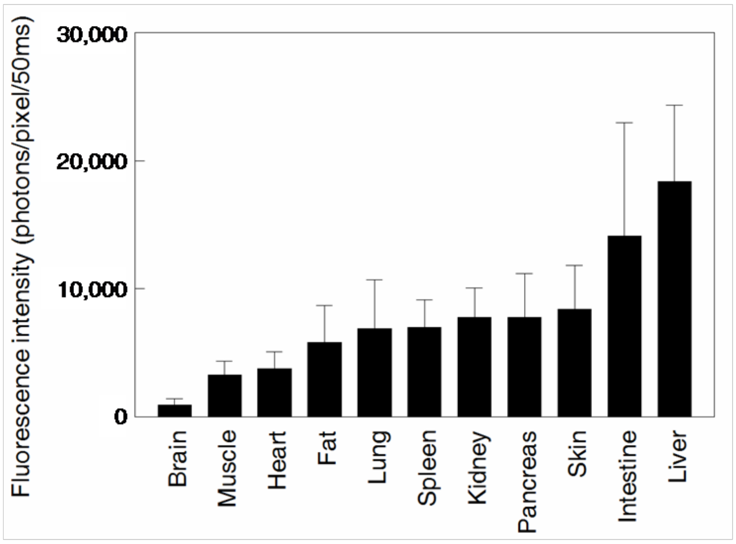

B

	Tumor	n	Fluorescence intensity (photons/pixel/50ms)			EPR
Subcutaneous	HUH-7	3	1,160	±	314	-
	HEK293(β3)	3	4,797	±	96	-
	IGROV1	4	12,247	±	2,292	+
	HT29	5	11,968	±	3,206	+
	TS/a-pc	3	15,311	±	5,177	+
	U87MG	3	12,034	±	2,061	+
Breast (Orthotopic)	TS/a-pc	6	15,160	±	5,561	+
Brain (Orthotopic)	U87MG	8	11,241	±	2,031	+

Supplementary Figure 4: Semi-quantifications of the quantity of fluorescent LNC 24 hours after intravenous injection in the different tumor models. The fluorescent intensities in organs (A) were similar among the different groups but varied largely depending on the tumor type (B). n: number of animals per group

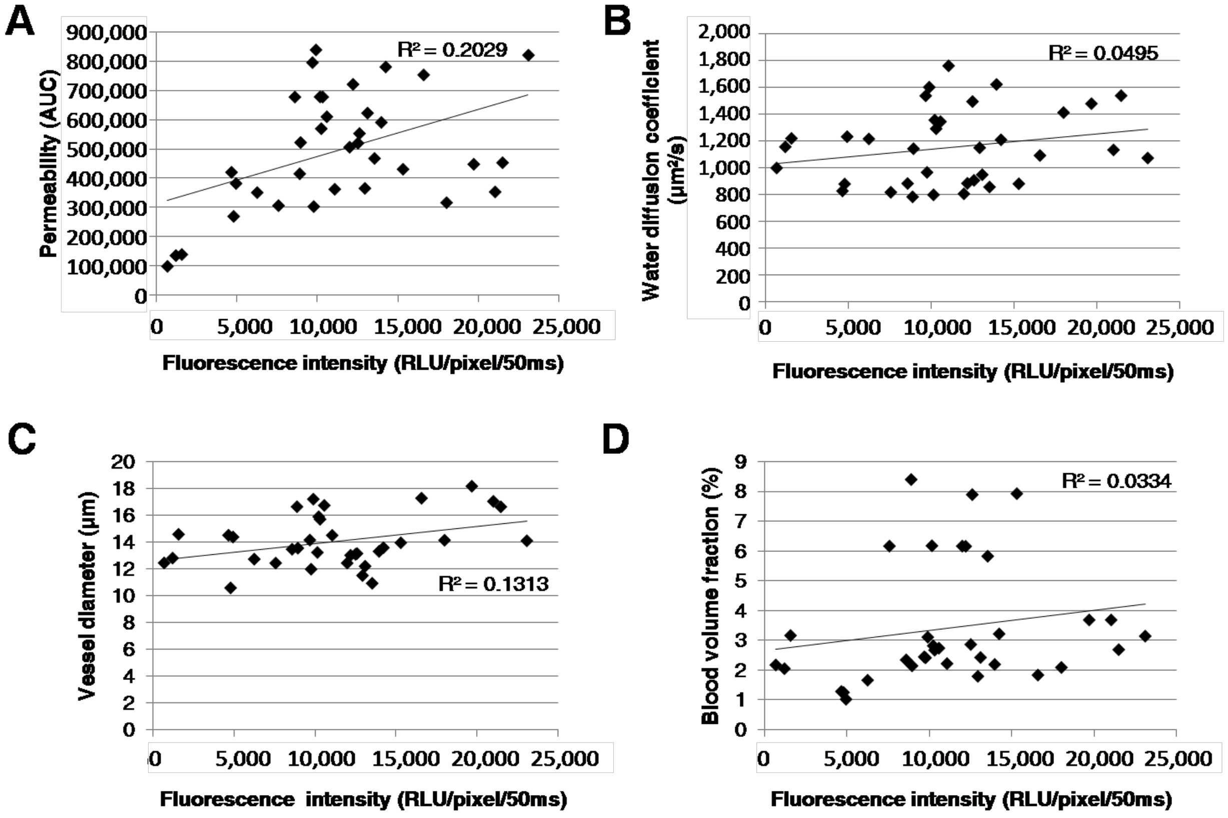

Supplementary Figure 5: Correlation between each tumor parameter evaluated by MRI and fluorescence imaging. Fluorescence intensities were compared to the permeability (A), water diffusion coefficient (B), vessel diameter (C) and blood volume fraction (D) obtained by MRI. The correlation factor R^2 was evaluated for each parameter.

A

$$Fluorescence = intercept + A \times AUC + C \times dia + \epsilon$$

AUC : permeability; dia : vessel diameter; Є = error

B

	Estimated parameter values	P
A	0.01 ± 0.004	0.0222
C	737 ± 451	0.1125

$$Fluorescence = - 4{,}319 + 0.01 \times AUC + 737 \times dia$$

$R^2 = 0.2642$

C

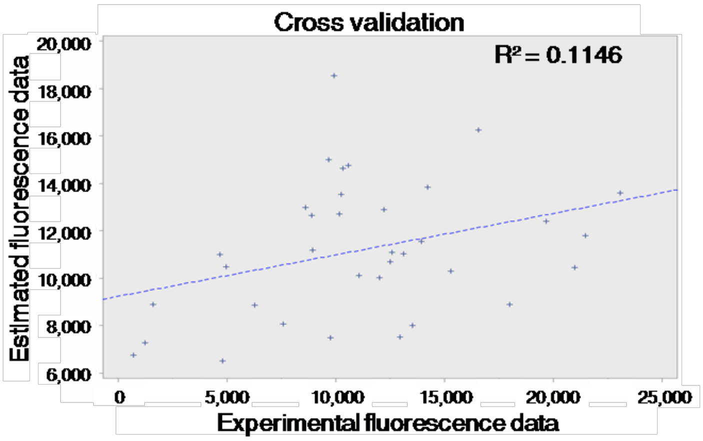

Supplementary Figure 6: Determination of a predictive equation between MRI parameters and fluorescence signals. An equation was determined using the SAS® software (A). Two of the four studied MRI parameters have a significant impact on the equation (B). The predictability of the equation is assessed by a cross validation test (C).
